# Exopolysaccharide Features Influence Growth Success in Biocrust-forming Cyanobacteria, Moving From Liquid Culture to Sand Microcosms

**DOI:** 10.3389/fmicb.2020.568224

**Published:** 2020-10-27

**Authors:** Sonia Chamizo, Alessandra Adessi, Giuseppe Torzillo, Roberto De Philippis

**Affiliations:** ^1^Department of Agronomy, University of Almería, Almería, Spain; ^2^Research Centre for Scientific Collections from the University of Almería (CECOUAL), Almería, Spain; ^3^Department of Agriculture, Food, Environment and Forestry (DAGRI), University of Florence, Firenze, Italy; ^4^CNR –Institute of BioEconomy, Sesto Fiorentino, Italy; ^5^Centro de Investigación en Ciencias del Mar y Limnología, Universidad de Costa Rica, San José, Costa Rica

**Keywords:** cyanobacteria liquid culture, sand inoculation, sandy soil microcosms, EPS monosaccharidic composition, EPS molecular weight distribution, semiarid soil

## Abstract

Land degradation in drylands is a drawback of the combined action of climate change and human activities. New techniques have been developed to induce artificial biocrusts formation as a tool for restoration of degraded drylands, and among them soils inoculation with cyanobacteria adapted to environmental stress. Improvement of soil properties by cyanobacteria inoculation is largely related to their ability to synthesize exopolysaccharides (EPS). However, cyanobacterial EPS features [amount, molecular weight (MW), composition] can change from one species to another or when grown in different conditions. We investigated the differences in growth and polysaccharidic matrix features among three common biocrust-forming cyanobacteria (*Nostoc commune, Scytonema javanicum*, and *Phormidium ambiguum*), when grown in liquid media and on sandy soil microcosms under optimal nutrient and water, in controlled laboratory conditions. We extracted and analyzed the released EPS (RPS) and sheath for the liquid cultures, and the more soluble or loosely-bound (LB) and the more condensed or tightly-bound (TB) soil EPS fractions for the sandy soil microcosms. In liquid culture, *P. ambiguum* showed the greatest growth and EPS release. In contrast, on the sandy soil, *S. javanicum* showed the highest growth and highest LB-EPS content. *N. commune* showed no relevant growth after its inoculation of the sandy soil. A difference was observed in terms of MW distribution, showing that the higher MW of the polymers produced by *P. ambiguum* and *S. javanicum* compared to the polymers produced by *N. commune*, could have had a positive effect on growth for the first two organisms when inoculated on the sandy soil. We also observed how both RPS and sheath fractions reflected in the composition of the soil TB-EPS fraction, indicating the role in soil stabilization of both the released and the cell attached EPS. Our results indicate that the features of the polysaccharidic matrix produced by different cyanobacteria can influence their growth success in soil. These results are of great relevance when selecting suitable candidates for large-scale cyanobacteria applications in soil restoration.

## Introduction

Cyanobacteria are the oldest oxygenic photosynthetic organisms. They are widespread in aquatic and terrestrial ecosystems, and occupy almost every habitat on Earth thanks to their ability to adapt to a wide range of environmental conditions (Whitton and Potts, 2002). In dryland soils, cyanobacteria are found in close associations with other organisms such as bacteria, algae, lichens and mosses forming the so called biological soil crusts or biocrusts (Weber et al., 2016). As part of these communities, cyanobacteria play key roles in soil properties and functions. Cyanobacteria filaments bind soil aggregates and create a stable surface layer that facilitates the path for colonization by other biocrust organisms such as lichens and mosses (Deng et al., 2020). Cyanobacteria fix CO_2_ (Miralles et al., 2018) and some species are able to fix N_2_, increasing soil organic matter and nutrient content (Mager and Thomas, 2011). They also release a wide array of substances in the soil such as growth-promoting regulators, vitamins, amino acids, polypeptides, biotins, proteins, and sugars that contribute to soil fertility and act as biocontrol agents against plant pathogenic bacteria, fungi and micro-algae (Singh et al., 2016). Cyanobacteria have received special attention as bio-inoculants for ecological restoration of degraded lands (Rossi et al., 2017). Soil inoculation with cyanobacteria has been shown to lead to soil improvements in desertified natural soils (Park et al., 2017), mine (Muñoz-Rojas et al., 2018) and quarry substrates (Roncero-Ramos et al., 2019a, b), fire-affected soils (Acea et al., 2003; Chamizo et al., 2020) and agricultural lands (Maqubela et al., 2009). Exopolysaccharides (EPS) are among the most important compounds synthesized by cyanobacteria playing a vital role in soil functions. Cyanobacterial EPS consist of polymeric substances of high viscosity with varying biochemical composition and biophysical properties that are among the most structurally and functionally complex bacterial structures (Hill et al., 1994). These biopolymers form an envelope surrounding the cells called sheath, glycocalyx, capsule, or slime, depending on its consistency and localization (De Philippis and Vincenzini, 1998), which protect cells from physical and biological stresses (Costa et al., 2018).

In dryland soils, cyanobacterial EPS are implied in soil stabilization, nutrient provision and resistance to desiccation (Brüll et al., 2000; Hu et al., 2003; Mager and Thomas, 2011). Exopolysaccharides regulate the loss and uptake of water from cells (Potts, 1994; Mazor et al., 1996; Adessi et al., 2018) and protect cells from damage during swelling and shrinkage due to frequent desiccation-rehydration cycles (Liu et al., 2017). After rewetting, cyanobacteria can rapidly recover metabolic activities and repair cellular components (Billi and Potts, 2002). Cyanobacterial EPS also contain sunscreen pigments that protect cells against UV-A/B radiation (Scherer et al., 1988; Ehling-Schulz et al., 1997; Fleming and Castenholz, 2007). More soluble soil EPS fractions or loosely-bound (LB) EPS are thought to represent an important source of energy for heterotrophic activity, while more condensed soil EPS fractions or tightly-bound (TB) EPS are mainly involved in soil particle consolidation, contributing to soil stability (Chen et al., 2014; Chamizo et al., 2019). Consequently, changes in soil properties can be related, to a large extent, to soil polysaccharidic matrix features such as amount, molecular weight (MW) distribution and chemical composition of soluble and condensed EPS fractions (Chen et al., 2014; Colica et al., 2014; Mugnai et al., 2018a, b; Chamizo et al., 2019). In general, EPS synthesis by a given cyanobacterial strain mostly depends on the species and the cultivation conditions such as the source of nitrogen, light intensity, temperature, salinity, and phosphorus and potassium contents (De Philippis and Vincenzini, 1998; Nicolaus et al., 1999; Otero and Vincenzini, 2003). In the soil, EPS characteristics have been related to the soil type (Chamizo et al., 2019) and biocrust age or successional stage (Chen et al., 2014; Colica et al., 2015).

Cyanobacterial EPS properties may represent an important factor to be considered for the selection of suitable cyanobacteria candidates for soil restoration. Cyanobacterial EPS characterization has been mainly done in liquid cultures for isolated strains but less explored in cyanobacteria-inoculated soil trials. Besides, little is known of how EPS features might change for a specific strain when grown in liquid culture and in the soil. The paucity of information may due to the fact that it is still not clear whether the soluble fraction of the EPS in liquid cultures is the same soluble fraction than when the strain grows in soil, similarly for the sheath EPS and the more strongly attached soil EPS. Another important factor to take into account for selection of adequate cyanobacteria bio-inoculants is that some strains might exhibit a fast growth when cultured in liquid conditions but limited growth when inoculated on the soil and vice versa. Thus, viability for biomass growing of the selected cyanobacteria in liquid culturing systems as well as capability to successfully colonize the soil after inoculation are key issues to be considered, especially for scaling up of cyanobacteria applications with restoration purposes. Last, the inoculated cyanobacteria should be able to induce significant improvements in soil properties and functions, so that the desired objective of soil recovery is fulfilled. In this respect, it would be very useful to have some descriptors of the potential of the strains for successful restoration approaches before actually applying them to the soil. Characterization of cyanobacterial EPS features in terms of their macromolecular distribution and monosaccharidic composition could provide valuable insights to this regard.

The main goal of this study was to examine whether different biocrust cyanobacterial strains showed contrasting performances when grown in liquid and solid medium and if so, if such differences could be linked to their EPS features. To achieve this goal, we analyzed the growth and polysaccharidic matrix features (amount, MW distribution and monosaccharidic composition) of three biocrust-forming cyanobacteria (*Nostoc commune, Scytonema javanicum*, and *Phormidium ambiguum*) when grown in liquid medium and after inoculation on a sandy soil under comparable optimal water and nutrient availability conditions. This can also help improve our understanding on how cyanobacterial performance and EPS traits might change when cyanobacteria are inoculated together with the nutrient media during their culturing, as a viable approach to improve biocrust performance and soil restoration success.

## Materials and Methods

### Selection of Cyanobacteria Strains

**FIGURE 1:**
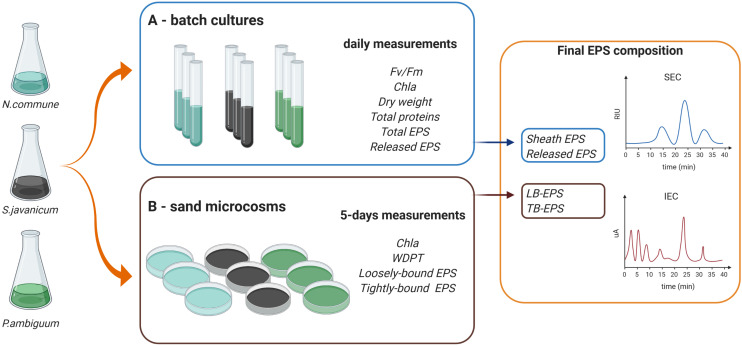
Scheme of the experimental design and variables measured in the **(A,B)** batch culture and sand microcosm experiments.

We selected three terrestrial cyanobacterial strains available in the laboratory collection and isolated from biocrust samples: the N-fixing *Nostoc commune*, belonging to the order Nostocales, isolated from the Negev desert; the N-fixing *Scytonema javanicum* Bornet & Flahault NIES-1956, belonging to the order Nostocales, originally isolated from the Tsukuba Botanical Garden (Japan); and the non N-fixing *Phormidium ambiguum* Gomont NIES-2121, belonging to the order Oscillatoriales, originally isolated from an African soil. These three genera have been described as part of the cyanobacterial community in biocrusts from semiarid regions (Roncero-Ramos et al., 2020). Besides, *N. commune* has been reported in biocrusts worldwide (Büdel et al., 2016). *Nostoc* spp., *Scytonema* spp. and *Phormidium* spp. have been also employed as soil inoculants to reverse land degradation and promote soil recovery in degraded arid soils (Li et al., 2014; Park et al., 2017; Zhao et al., 2019). The selected cyanobacterial strains were used for the batch culture and sand-microcosm experiments described below. A scheme summarizing the experimental design and the variables measured on each experiment can be seen in Figure 1.

### Batch Culture Experiments in Liquid Media

Biomass of the selected cyanobacterial strains was collected and used to set up the batch culture experiments (Figure 1A). The strains were transferred to aerated glass vertical columns (diameter 50 mm) with a working volume of 500 ml. BG-11_0_ medium (Rippka et al., 1979) was used for the heterocystous strains, *N. commune* and *S. javanicum*, and BG-11 medium (with nitrate) for the non-heterocystous strain, *P. ambiguum*. The columns were placed in a thermostated water bath at a constant temperature of 30°C and were continuously illuminated on both sides, with a photon flux density (PFD) of 70 μmol m^–2^ s^–1^. The strains were acclimated to these conditions during two weeks previous to perform growth curve measurements. Cultures were bubbled with filter-sterilized air supplemented with CO_2_ (0.05%v/v.) in order to homogenize the culture and keep the pH close to 7.0. Three replicates were considered for *N. commune* and four replicates for *S. javanicum* and *P. ambiguum* due to their frequent cell aggregation which introduced higher variability of parameter measurements. Cultures were inoculated at an initial dry weight of 0.1 g L^–1^ and the growth curve for the three strains was followed for 9 days. Evaporation was compensated daily with sterilized distilled water. Every day, a sample of 30 mL was withdrawn from each culture to monitor the culture behavior and analytical measurements.

#### Dry Weight and Chlorophyll a Concentration

Dry weight was measured by filtering 5–10 mL of culture on a pre-weighted filter (Whatman grade GF/F) 1 μm pore filters, and then dried at 105°C for 3 h. Chlorophyll *a* concentration was measured following the method by Singh et al. (2016). In brief, 5 mL of culture was centrifuged at 2500 × *g* at laboratory temperature for 7 min and the supernatant was thoroughly discarded, after which 5 mL of methanol was added and the sample was vortexed and heat at 70°C. Then, samples were centrifuged at 2500 × *g* and absorbance of the supernatant was measured in a spectrophotometer at 665 nm and 750 nm. Chlorophyll *a* content was determined according to the equation by Ritchie (2006).

Chla [μg/ml] = 12.9447 (A665 – A750) x Volume of methanol (mL)/Volume of sample (mL) (Eq. 1).

#### Fluorescence Parameters

The ratio between variable and maximum fluorescence, F_v_/F_m_, was measured to determine the maximum photochemical quantum yield of PSII, using a pulse-amplitude-modulation fluorimeter (PAM-2100, H. Walz, Germany). For this purpose, samples were taken from the cultures and incubated in the dark for 15 min to remove any energy-dependent quenching. Then, one far-red light (above 700 nm) pulse with a duration of 10 sec (10 W m^–2^), supplied by the PAM-2100, was applied. This procedure was applied to attain a full oxidation of the plastoquinone pool (PQ). For comparison, measurements of F_v_/F_m_ were also performed in the light using 3-(3,4-dichlorophenyl)-1,1-dimethylurea (DCMU) (10^–5^ M), and resulted in a lower value, therefore all the measurements were carried out using far red light. In addition, rapid light-response curves (RLCs) of cultures were measured daily using a 2 mL cell sample placed in a Liquid-Phase Oxygen Electrode Chamber (Hansatech, DW3) cuvette, thermostated at 25°C. A series of stepwise increasing irradiance intensities (LEDs, 0–636 μmol photons m^–2^ s^–1^) provided by PAM-2100 were automatically applied at 20 s intervals to obtain the light-adapted fluorescence level F’ (steady-state fluorescence yield in the light), and at the end of each step a saturating pulse (>6,000 μmol photons m^–2^ s^–1^, 0.6 s duration) was triggered to reach the maximum fluorescence level F_m_’ (steady state maximum fluorescence in the light). The effective PSII photochemical quantum yield in the light, Y_*II*_, was determined as (F_m_’- F’)/F_m_’ in the light-adapted state at respective irradiance level. The effective quantum yield of PSII was used to calculate the electron transfer rate (ETR). However, it must be pointed out that with cyanobacteria, this parameter can furnish only a relative measure of the ETR, since the PSI/PSII ratio is much higher than in leaves (Vermaas, 2001; Fraser et al., 2013), and the light absorption coefficient can affect the measurements (Szabó et al., 2014). Relative electron transport rates (rETR = μmol e- m^–2^s^–1^) were calculated as rETR = PFD x (F_m_’- F’)/F_m_’ x ETR factor (i.e., the fraction of light absorbed by the sample and distributed to PSII). An ETR factor of 0.42 was used, which takes into account the default setting for percentage of light absorbed (0.84) and that distributed to PSII (0.5). Analysis of RLCs was used to calculate changes in important parameters, that is, the maximum relative electron transport rate through PSII, rETR_max_, the initial slope, α, of the rETR vs. PFD curve which is the quantum efficiency of the photosynthesis, the saturation irradiance, I_k_, given as intercept between α, and rETR_max_. The curves were fitted to the non-linear least-squares regression model by Eilers and Peeters (1988) using PamWin 3 software.

#### EPS Characterization

Total EPS content and released EPS (RPS hereafter) were also daily determined in the cultures. For total EPS content, 1 mL of the culture was taken and its carbohydrate content was quantified by means of the phenol-sulfuric acid assay (Dubois et al., 1956). RPS were extracted by centrifuging 5 mL of culture at 4000 × *g* for 30 min. Then, the supernatant was recovered and an aliquot of 1 mL was taken to quantify carbohydrate content using the phenol-sulfuric assay. When EPS amount was very high and/or there was interference of nitrate in BG-11 cultures, a 1:5 or 1:10 dilution was applied to the samples; v) Protein content of the cultures was measured by the Lowry method using bovine serum albumin as standard (Lowry et al., 1951).

Additionally, at the end of the growth curve, apparent molecular weight (MW) distribution and monosaccharidic composition of the cyanobacterial sheath and RPS of the three strains were determined. For this, the remaining culture of the strains at the end of the experiment and once the described measurements were done, was mixed and centrifuged to separate the RPS from the cell biomass. Then, the cyanobacterial sheath was recovered from the pellet by washing cyanobacterial cells with 5 mL of distilled water at 80°C for 1 h, centrifugation at 4000 × *g* for 30 min and recovery of the supernatant. This operation was repeated three times. In the cases in which the sheath was strongly attached to cells (e.g., *P. ambiguum*), the sheath-containing pellet was additionally mixed with a 1.5% NaCl solution and then extracted with 5 mL of distilled water at 80°C for 1 h (Rossi et al., 2018). Effective sheath extraction from cyanobacterial filaments was checked by optical microscope observations. MW distribution of RPS and cyanobacterial sheaths was determined by size exclusion chromatography (SEC). 1 mL of the sheath- and RPS-containing extracts was taken and injected in a Varian Pro-Star liquid chromatograph (Varian Inc., United States) equipped with a refractive index (RI) detector and two columns, PolySep-GFC-P 6000 and 4000 (Phenomenex, United States), connected in series. The columns (700 mm length and 7.8 mm internal diameter) had separation ranges of 100 kDa to 15 MDa and 0.3 to 400 kDa, respectively. Samples were analyzed with runs of 70 min using HPLC-grade water as eluent at a flow rate of 0.4 mL min^–1^. Size classes were established according to retention times of known MW dextran standards (Sigma-Aldrich, United States). These MW classes were: >2000 kDa (0–25 min), 2000–1100 kDa (25–35 min), 1100–410 kDa (35–36 min), 410–150 kDa (36–38 min), 150–50 kDa (38–40 min) and <50 kDa (40–70 min). To obtain the% of the different MW classes, we performed the ratio of each peak area to the total area under the curve and assigned the resulting% area to the corresponding size class according to the retention time of the peak output.

The monosaccharide composition was analyzed using Ion Exchange Cromatography (IEC) following the procedures described in Mugnai et al. (2018a, b) and Chamizo et al. (2019). Before IEC analysis, 1 mL of the extracts (sheath or RPS) was mixed with 1 mL of 4 N trifluoroacetic acid (TFA) and heated for 120 min at 120°C. Afterward, the excess of TFA was removed by drying on a rotary evaporator and the dried extracts re-solubilized in deionized water, repeating this operation three times per sample. Monosaccharide composition was analyzed with a Dionex ICS-2500 ion exchange chromatograph (Dionex, United States) equipped with an ED50 pulsed amperometric detector operating with a gold working electrode (Dionex) and a CarboPac PA1 column of 250-mm length and 4.6-mm internal diameter (Dionex). We used as eluents HPLC-grade water (A), 0.185 M Na hydroxide (B), and 0.488 M Na acetate (C), at a flow rate of 1 mL min^–1^. Single sugars were identified on the basis of the retention time of known standards. Results were expressed as molar ratio.

### Sandy Soil Microcosms Experiments

#### Preparation and Inoculation of the Sandy Soil Microcosms

Cultures of the same cyanobacterial strains used for the batch culture experiments were used for the sand-microcosms experiment (Figure 1B). The sandy soil (92% sand, 1% silt and 7% clay) was collected from a semiarid area in Almeria province (SE Spain) and had an organic carbon content of 1.23 g kg^–1^ and nitrogen content of 0.21 g kg^–1^. Small Petri dishes (12 mm height x 54 mm diameter) were filled with 30 g of sterilized sand (by autoclaving twice for 20 min at 120°C). Inoculation was done by adding 30 mg (dry weight) of cyanobacterial biomass on each Petri dish or sand microcosm, equivalent to 5 g m^–2^, which has been found to be an optimal areal density to promote biocrust formation on sandy soils (Mugnai et al., 2020). In addition, chlorophyll *a* content of the inoculum solutions was measured following the procedure explained above for the liquid culture. Chlorophyll *a* concentrations of *N. commune*, *S. javanicum*, and *P. ambiguum* solutions were 10.4 ± 1.5, 11.0 ± 2.3 and 8.1 ± 1.0 μg mL^–1^, respectively. Soil samples with non-inoculated sand were used as controls. Each treatment (control and sand inoculation with each strain) was done in triplicate. Sand microcosms were incubated in a plexiglass growth chamber with controlled temperature (30°C) and light intensity (70 μmol photons m^–2^ s^–1^) for 30 days. Water and nutrients were added to the microcosms in order to provide optimal conditions for cyanobacteria growth, similar to that of liquid cultures. Microcosms were watered with 5 mm of distilled water five days a week. In addition, 5 mL of culture media (3 x concentrated) (BG11 for the sand inoculated with the non-heterocystous strain and BG11_0_ for the sand inoculated with the heterocystous strains) was added to the soil samples every 5 days. The amount of nutrients applied to the soil after 30 days was equivalent to 0.5 L of culture media (similar to that of the liquid cultures). Addition of water and nutrients was also done on the control samples. Of the three control replicates, two were sprayed with BG11 and one with BG11_0_. As no difference was found in the control sand provided either with BG11 or BG11_0_, the average of the three replicates was considered for further comparisons with the inoculated sand treatments. Six samplings were carried out during the experimental period, so that a total of 72 samples (4 treatments x 3 replicates x 6 samplings) were prepared.

#### Soil Determinations

Every 5 days, 12 samples (three per treatment) were randomly selected and the surface crust (2 mm thick) was collected in order to determine chlorophyll *a* content, as a proxy of cyanobacterial growth, and EPS content. Previous to crust sampling, soil hydrophobicity was measured on the sand microcosms using the water drop penetration time (WDPT) test (Doerr, 1998). Five to seven drops of distilled water were dropped from a height of 1.5 cm on to the surface of the soil sample. The average time the drops remained on the surface was used as an index of the severity of water repellency. After this test, the crust was collected with a small putty knife and ground to a fine powder with mortar and pestle. Chlorophyll *a* content was determined after extraction with hot ethanol at 80°C for 5 min, centrifugation and measurement of supernatant absorbance at 665 nm (Castle et al., 2011). Chlorophyll *a* content was calculated according to Ritchie (2006) equation:

Chlorophyll *a* = (11.9035 × A665_0_ × V) x (g soil^–1^) x L (Eq. 2)

where V is the volume of solvent (mL) and L is the path length.

#### Soil EPS Characterization

The amount of two soil EPS fractions were determined in the crust: the more soluble and less condensed fraction, easily released into the environment or “loosely bound EPS” (referred to as LB-EPS), and the more condensed fraction, firmly attached to the cells and soil particles or “tightly bound EPS” (referred to as TB-EPS). LB-EPS were extracted with distilled water at room temperature for 20 min. The supernatant was recovered after centrifugation at 3500 × *g* for 30 min. This process was repeated three times for each sample and the three supernatants obtained were mixed together. TB-EPS were recovered from the resulting pellet using three extractions with 0.1 M Na_2_EDTA and centrifugation at 3500 × *g* for 30 min. The three supernatants obtained after the three extractions were mixed together. The carbohydrate content of both LB-EPS and TB-EPS extracts was determined using the phenol-sulfuric acid assay.

At the end of the experiment (30 days), the apparent MW distribution and monosaccharide composition of the two soil EPS fractions were also determined. In the TB-EPS fraction, the excess of Na_2_EDTA that could interfere with the chromatographic analysis was removed by dialyzing the extracts in nitrocellulose tubular membranes (14 kDa MW cutoff, Medicell International, United Kingdom) for 24 h in distilled water. Extracts were then dried and dissolved in deionized water, transferred to Eppendorf tubes, and clarified by ultracentrifugation at 13,000 × *g* in order to remove the coarse particulate. The MW distribution was analyzed by size exclusion chromatography (SEC) following the procedure explained above for the liquid culture extracts. For monosaccharidic composition determination, the extracts were hydrolyzed and purified following the same procedure explained above for the liquid culture and monosaccharide composition was determined by IEC analysis following the previously described methodology.

### Data Elaboration and Statistical Analysis

Differences in dry weight, chlorophyll *a* content, fluorescence parameters, and total and released EPS content among the three cyanobacterial strains grown in liquid culture were analyzed using one-ANOVA and the Tukey *post hoc* test. Variables were previously checked for normality and homogeneity of variance using the Shapiro-Wilk and Levene’s test. When needed, data were log transformed before performing parametric analysis. In the case of the sandy soil microcosms, as variables did not meet normality assumptions, the effect of the inoculation treatment (control and sand inoculation with the three strains) and incubation time on chlorophyll *a*, LB-EPS and TB-EPS was analyzed using a permutational multivariate analysis of variance (PERMANOVA) based on Euclidean distances. Further differences among the strains were analyzed with paired-wise tests using a maximum of 9999 permutations and applying Monte Carlo correction. All the analyses were performed using Primer 7 and Permanova + (PRIMER-E Ltd., Plymouth, United Kingdom). The complexity of the monosaccharidic profiles was analyzed and interpreted calculating diversity indices. Thus, alpha-diversity of sugar residues of the different strains and conditions (sheath and RPS in liquid cultures, and LB-EPS and TB-EPS in the sand microcosms) were compared. Diversity indexes were calculated using the percentiles of a bootstrap distribution with 9999 repetitions, using the Past 4.0 software. Significance was established at *p* < 0.05.

## Results

### Cyanobacteria Growth in Liquid Cultures

**FIGURE 2:**
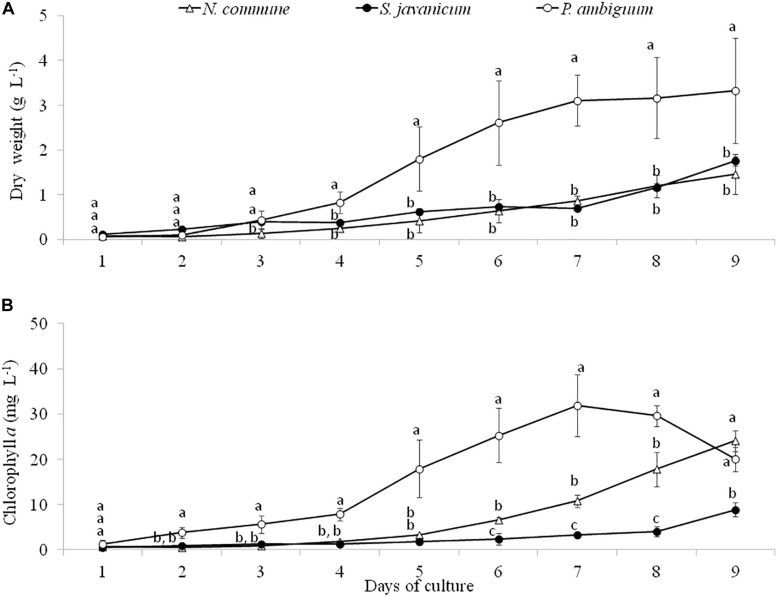
Dry weight **(A)** and chlorophyll *a* concentration **(B)** during the cultivation period of the three cyanobacteria strains. Different letters indicate significant differences among the strains for each day.

Batch cultures of the three cyanobacteria strains were monitored during 9 days through determination of dry weight and chlorophyll *a* concentration. The greatest growth was observed with *P. ambiguum* which reached a dry weight of 3.33 g L^–1^ after 9 days, while *N. commune* and *S. javanicum* showed a lower growth, 1.46 g L^–1^and 1.75 g L^–1^, respectively (Figure 2A). The higher growth showed by *P. ambiguum* was also reflected in a higher chlorophyll *a* increase, which reached 31.9 mg L^–1^ at day 7 and then progressively decreased until day 9 (Figure 2B). Chlorophyll *a* concentration increased in *N. commune* and *S. javanicum* over time and after 9 days of biomass culturing, reached values of 24.1 mg L^–1^ and 8.9 mg L^–1^, respectively (Figure 2B). Chlorophyll increase with time showed a Gompertz curve fitting for *P. ambiguum* (r^2^ = 0.9903) and an exponential curve for *N. commune* and *S. javanicum* (r^2^ = 0.9667 and 0.9901, respectively). Protein content of the cultures at the end of the experiment reached 796.0 ± 135.5 mg L^–1^ for *N. commune*, 933.4 ± 306.1 mg L^–1^ for *S. javanicum* and 1128.2 ± 290.3 mg L^–1^ for *P. ambiguum*, representing, respectively, 55%, 53% and 34% of their respective dry weight.

**FIGURE 3:**
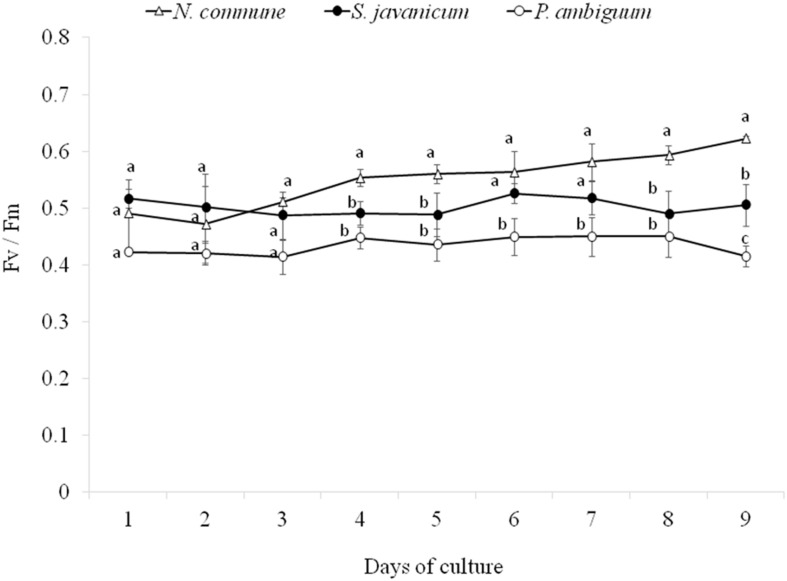
Time course of the daily values of the F_*v*_/F_*m*_ ratios measured during the cultivation period of the three cyanobacteria strains (mean ± SD, *n* = 9). Different letters indicate significant differences among the strains for each day.

During the whole period of culture, the F_*v*_/F_m_ ratio remained rather constant or slightly increased in *N. commune*, indicating good physiological state of the cultures (Figure 3). The mean values of the F_v_/F_m_ were 0.525 ± 0.039 in *N. commune*; 0.502 ± 0.014 in *S. javanicum*, and 0.434 ± 0.016 in *P. ambiguum* (Table 1). The mean values of the calculated parameters of RLCs, i.e., the rETR_*max*_, the initial slope (α) of the rETR vs. PFD curves, and the saturation irradiance (I_k_) are reported in Table 1. The mean rETR_max_ gathered over the whole cultivation period resulted about 25% higher in *P. ambiguum*, while the initial slope, i.e., the quantum efficiency of photosynthesis, was higher in *N. commune* (0.228) than in *S. javanicum* (0.190) and *P. ambiguum* (0.174). The highest saturation irradiance of photosynthesis was found in *P. ambiguum* (Table 1).

**TABLE 1:** Mean values of fluorescence parameters measured over a cultivation period of nine days in cultures of *N. commune, P. ambiguum, and S. javanicum*.

Fluorescence parameters	*Cyanobacteria strain*		
	*N. commune*	*S. javanicum*	*P. ambiguum*
rETR_max_ (μmol e- m^–2^ s^–1^)	40.85 ± 10.25^a^	40.0 ± 17.72^a^	52.80 ± 33.0^a^
I_k_ (μmol photons m^–2^ s^–1^)	178.62 ± 26.19^b^	211.42 ± 89.28^ab^	303.57 ± 32.68^a^
α (μmol e- μ photons^–1^)	0.228 ± 0.031^a^	0.190 ± 0.018^b^	0.174 ± 0.033^c^
F_v/_F_m_	0.525 ± 0.039^a^	0.502 ± 0.014^b^	0.434 ± 0.016^c^

*Data are the mean values ± SD (n = 9). Different letters indicate significant differences among the strains. rETR_max_: maximum relative electron transport rate; I_k_: saturation irradiance; α: initial slope of the rETR vs. PFD curve; F_v_/F_m_: ratio between variable and maximum fluorescence.*

**FIGURE 4:**
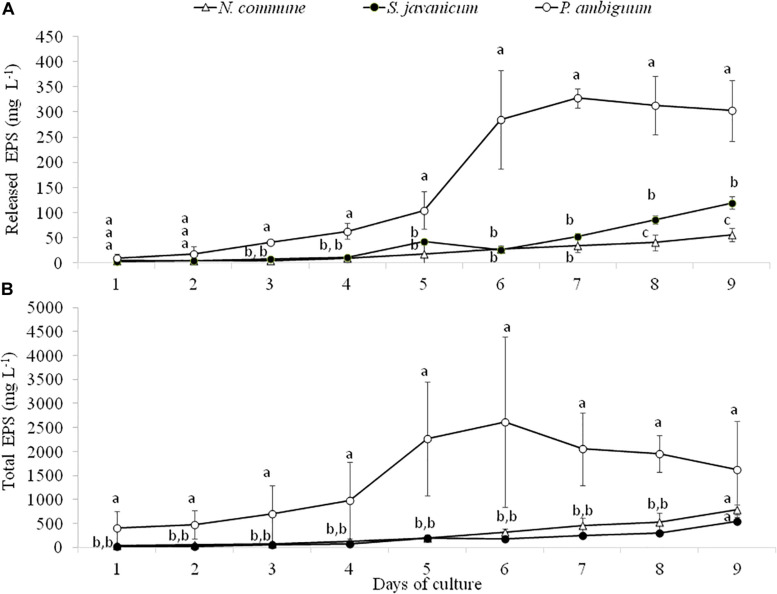
Released **(A)** and total EPS **(B)** content during the cultivation period of the three cyanobacteria strains. Different letters indicate significant differences among the strains for each day.

Analysis of EPS production during cyanobacteria growth showed a similar trend to chlorophyll content. The amount of released and total EPS was significantly higher in *P. ambiguum* showing the highest values at day 6 and 7, after which it slightly decreased in both EPS fractions (Figures 4A,B), in coincidence with the pattern of chlorophyll content. *N. commune* and *S. javanicum* showed lower EPS contents and significant differences were found between them in the released EPS. While the total EPS amount was similar for both cultures (Figure 4B), *N. commune* showed a higher amount of released EPS than *S. javanicum* (Figure 4A).

### Cyanobacteria Growth in Sandy Soil Microcosms

**FIGURE 5:**
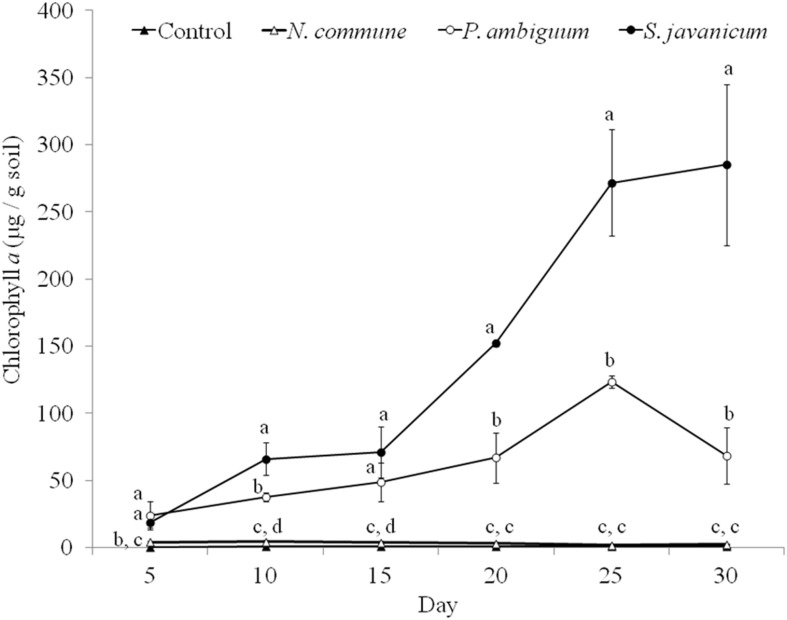
Chlorophyll *a* content over time in the control and inoculated sandy soil microcosms. Different letters indicate significant differences among the control and cyanobacteria-inoculated soils for each sampling day.

**FIGURE 6:**
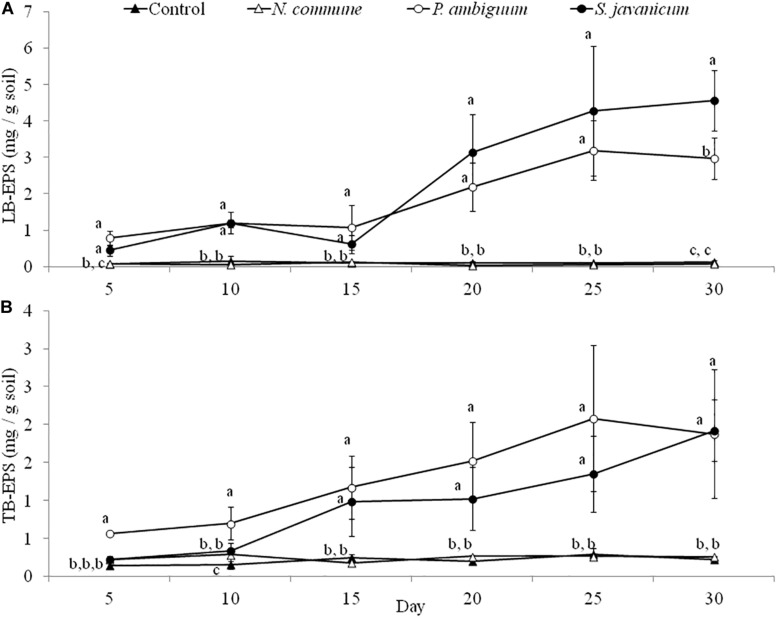
LB-EPS **(A)** and TB-EPS **(B)** contents over time in the control and inoculated sandy soil microcosms. Different letters indicate significant differences among the control and cyanobacteria-inoculated soils for each sampling day.

The cyanobacteria strain and incubation time, as well as interaction between them, had a significant effect on chlorophyll *a* content and LB-EPS and TB-EPS amounts (*p* < 0.05), indicating that change of these variables with time depended on the strain. Chlorophyll *a* content increased in the inoculated sand with *P. ambiguum* and *S. javanicum*, thus indicating a significant cyanobacterial growth over time. However, no significant increase was observed in the inoculated sand with *N. commune*, which showed a chlorophyll content close to zero and similar to control soils (Figure 5). In contrast to the pattern observed in batch cultures where *P. ambiguum* exhibited the highest growth, in the sand microcosms *S. javanicum* was the strain that showed a better performance. After 30 days of soil incubation, chlorophyll content resulted four times higher in the *S. javanicum-*inoculated sand than in the *P. ambiguum*-inoculated sand. The content of the two soil EPS fractions differed between the two strains. LB-EPS content was similar in the inoculated sand with *P. ambiguum* and *S. javanicum* during the first days after sand inoculation (Figure 6A) but it was significantly higher in *S. javanicum* from the fifteenth day onward (Figure 6A), also coinciding with a sharper increase in chlorophyll content. TB-EPS content increased in both inoculated soils over time and although the sand inoculated with *P. ambiguum* showed higher TB-EPS content than the sand inoculated with *S. javanicum*, the difference resulted not significant (Figure 6B). It is worth mentioning that a significant increase in soil hydrophobicity was found in the sand inoculated with *S. javanicum* from day 10 till the end of the experiment. While controls and the other inoculated soils showed no water repellence (WDPT < 5s), *S. javanicum*-inoculated sand showed WDPT > 60 s in most cases and some times lasting for several minutes, indicating severe hydrophobicity.

### EPS Molecular Weight Distribution and Monosaccharidic Composition of the Cyanobacterial Strains in Liquid Culture and Soil Microcosms

**FIGURE 7:**
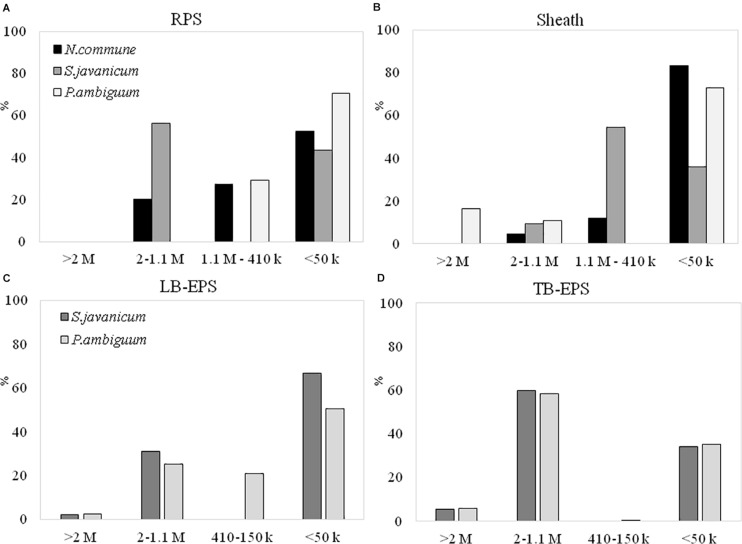
MW distribution of the EPS extracted from liquid culture: **(A)** RPS, **(B)** sheath, and from sandy soil microcosms: **(C)** LB-EPS; **(D)** TB-EPS, of the cultured strains. Relative proportions (%) of MW classes are represented. No data are reported for *N. commune* in the sandy soil microcosms (**C,D**) because of the negligible amounts of EPS extracted (See Figure 5).

**FIGURE 8:**
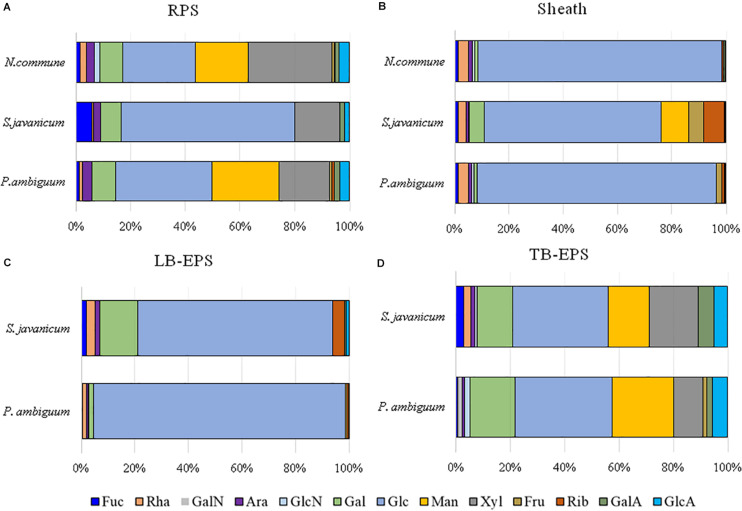
Monosaccharidic composition of the EPS extracted from liquid culture: **(A)** RPS, **(B)** sheath, and from sandy soil microcosms: **(C)** LB-EPS; **(D)** TB-EPS, of the cultured strains. Molar percentages (%) of single sugars are represented (expressed as moles of the single monosaccharide divided by the total amount of moles of monosaccharides in the EPS × 100). No data are reported for *N. commune* in the sandy soil microcosms (**C,D**) because of the negligible amounts of EPS extracted. Abbreviations: Fuc fucose, Rha rhamnose, GalN galactosamine, Ara arabinose, GlcN glucosamine, Gal galactose, Glc glucose, Man mannose, Xyl xylose, Fru fructose, Rib ribose, GalA galacturonic acid, GlcA glucuronic acid.

The EPS extracted both from culture biomass and from the soil microcosms were analyzed in terms of molecular weight (MW) distribution and monosaccharidic composition. The EPS extracted from *N. commune* inoculated microcosms were insufficient for these in-depth analytical purposes (see Results in Figure 6), hence data are not shown in Figures 7C,D, 8C,D. The MW distribution results (Figure 7) showed that the polymers were composed of four classes of MW, from >2MDa to <50 kDa, not equally represented among strains and among culturing conditions. In liquid cultures the highest MW class (>2MDa) could only be detected in *P. ambiguum* sheath (16.3%), while the lowest MW class (<50kDa) was always largely represented (ranging from 36.0% in *S. javanicum* sheath to 83.4% in *N. commune* sheath) (Figure 7B). The released EPS were mostly composed of low MW molecules <50KDa. However, *S. javanicum* showed also a high percentage of the MW class 2MDa-1.1MDa (56%), as well as *N. commune* which showed high percentages of the MW class 2MDa-1.1MDa (20%) and 1.1MDa–410 kDa (27%) (Figure 7A).

In the sandy soil microcosms (Figures 7C,D), the EPS MW distribution profiles of the two strains were similar, either for LB-EPS or TB-EPS: the only exception was the presence of the intermediate MW class (1.1MDa–410 kDa) in *P. ambiguum* LB-EPS. The highest MW class (>2MDa) was always represented both in LB-EPS (2.5 and 2.1% for *P. ambiguum* and *S. javanicum*, respectively) and in TB-EPS (6.0 and 5.7% for *P. ambiguuum* and *S. javanicum*, respectively), while the lowest MW class (<50kDa) was more represented in LB-EPS (50.5 and 67.0% for *P. ambiguum* and *S. javanicum*, respectively) than in TB-EPS (35.1 and 34.3% for *P. ambiguum* and *S. javanicum*, respectively).

The EPS monosaccharidic composition was heterogeneously dependent on the strain or on the culturing conditions (Figure 8, Supplementary Table S1). In almost all cases the most represented sugar residue was glucose, but many differences could be observed. Due to the complexity of the monosaccharidic profiles and the number of conditions to compare, for a clearer and informative interpretation of analytical data, we used an innovative approach for the description of monosaccharidic composition, as usually done in ecologic approaches, based on analysis of diversity indices of the samples. The results referring to the liquid cultures (Figures 8A,B, Table 2, and Supplementary Table S1) showed similar profiles of *N. commune* and *P. ambiguum* in terms of most represented sugars (a 3% threshold was arbitrarily set to define a significantly represented sugar): glucose, mannose, xylose, galactose, arabinose and glucuronic acid, in order from the most to the less represented residue in RPS; besides glucose, only rhamnose was present (>3%) in the sheath. On the contrary, *S. javanicum* showed glucose, xylose, galactose and fucose as the main RPS components, and glucose, mannose, ribose, fructose, and galactose composing the sheath. In general, the RPS composition showed a higher diversity than sheath composition, but some differences were observed among the strains. *S. javanicum* maintained the same diversity between RPS and sheath, while *P. ambiguum* and *N. commune* showed a lower diversity for the sheath compared to the RPS (Table 2).

**TABLE 2:** Diversity indices of the EPS fractions extracted from the three strains in the two culturing conditions.

Culture condition	EPS fraction	Strain	Number of sugar residues S	Dominance D	Shannon H	Evenness e^H/S	Equitability J	Fisheralpha
Liquid	RPS	*N. commune*	12	0.21	1.81	0.51	0.73	1.35
culture		*S. javanicum*	8	0.44	1.21	0.42	0.58	0.85
		*P. ambiguum*	11	0.23	1.74	0.52	0.72	1.22
	Sheath	*N. commune*	9	0.82	0.50	0.18	0.23	0.98
		*S. javanicum*	11	0.45	1.28	0.33	0.53	1.22
		*P. ambiguum*	11	0.44	0.59	0.32	0.52	1.22
Sandy soil	LB	*S. javanicum*	7	0.56	0.95	0.37	0.49	0.74
microcosms		*P. ambiguum*	8	0.88	0.33	0.17	0.16	0.89
	TB	*S. javanicum*	10	0.21	1.84	0.63	0.80	1.10
		*P. ambiguum*	11	0.23	1.75	0.50	0.72	1.28

In the sandy soil microcosms, comparison between *P. ambiguum* and *S. javanicum*-inoculated soils highlighted differences mostly in LB-EPS composition, while TB-EPS composition resulted similar between the two strains (Figures 8C,D, Supplementary Table S1). Glucose was the most abundant monosaccharide in LB-EPS and also galactose was found at a relatively high percentage in *S. javanicum-*inoculated sand (Figure 8C). TB-EPS was mostly composed of glucose, galactose, mannose, xylose, and uronic acids in both inoculated soils (Figure 8D, Supplementary Table S1). Noticeably, the monosaccharidic profile composing TB-EPS resulted similar or even higher than the respective sheath or RPS profiles, in terms of diversity (Table 2). In *P. ambiguum*-inoculated sand, mannose and xylose were present in TB-EPS, and the same were only present in RPS (and not in the sheath); same occurred for xylose in *S. javanicum*-inoculated microcosms. On the other hand, the TB-EPS extracted from *S. javanicum*-inoculated microcosms contained for example mannose, which was only present in the strain’s sheath (and not in its RPS).

## Discussion

The current study allowed exploring the growth performance and amount and chemical features of EPS of three biocrust cyanobacterial strains when grown in liquid culture and on a sandy soil. Although direct extrapolation of results from one experimental setting to another cannot be done due to the intrinsic characteristics of the growing media (liquid and solid), which affect light distribution, cyanobacteria dispersion in the medium, and contrasting capabilities for the use of nutrients, among others, application of similar conditions for both experiments (light, temperature and addition of nutrient medium) facilitates, to the best extent, comparisons of results among strains in the two growing conditions. Contrasting growth performances and EPS synthesis and chemical features of the cyanobacterial strains under the two growth conditions provide valuable insights for the screening of cyanobacteria candidates to be used in soil restoration.

### Growth and EPS Characteristics of the Cyanobacterial Strains in Liquid Cultures

The three cyanobacteria strains showed a significant growth within 9 days, as shown by the increase of dry weight and chlorophyll concentration. At the end of the experiment, the dry weight surpassed 1 g L^–1^ in all the strains (Figure 2). *P. ambiguum* was the strain that showed the higher growth, reaching 3.33 g L^–1^ (dry weight). F_v_/F_m_ remained stable during the whole cultivation period although at different levels. The highest value (0.525 ± 0.039) was found in *N. commune* (Figure 3). The F_v_/F_m_ ratios, usually lower in cyanobacteria than in green algae, can be explained by different ratios of PSI/PSII, which in cyanobacteria varies from 2 to 3.5 (Vermaas, 2001; Fraser et al., 2013), and raises the level of minimum level of fluorescence, Fo. However, F_v_/F_m_ ratios well mirrored those of initial slopes (α) in the three tested strains. The rETR values of the strains correlated well with the increase in biomass being higher in *P. ambiguum*. This cyanobacterium showed the lowest quantum yield and the highest saturation irradiance I_k_ (Table 1), meaning that this organism utilizes light with lower efficiency and requires more light to saturate photosynthesis. One consequence could be that this cyanobacterium, compared to *N. commune* and *S. javanicum* is more adapted to cope with high light exposure, a condition that could be found in arid zones.

In all the strains the increase in cyanobacterial biomass over time was accompanied by a parallel increase in both total EPS (cellular EPS + RPS) and RPS contents (Figure 4). An increase in the amount of RPS along with cell growth in batch cultures has been previously documented (Rossi and De Philippis, 2015). Total EPS content was up to 14 times higher than RPS content, highlighting the significant contribution of cell sugars content to total EPS amount. Parallelly to the higher dry weight and chlorophyll content in *P. ambiguum*, this strain also showed the highest amounts of total and released EPS. The greater growth and EPS synthesis by *P. ambiguum* in comparison to the other two strains can be attributed to the presence of a nitrogen source in the medium, which is a condition requiring lower energy for the assimilation of combined nitrogen compared with the energy needed for nitrogen fixation (Otero and Vincenzini, 2003).

In our experiments, with nutrient replete cultures, both RPS and sheath fractions showed the presence of very high MW polymers (> 1.1MDa). Previous studies have also confirmed that most cyanobacterial EPSs are characterized by the presence of high MW components (Pereira et al., 2009). However, a relevant presence of smaller macromolecules (<50kDa) was also found in both RPS and sheath (Figures 7A,B). This result is in contrast with that obtained by Mugnai et al. (2018a) who found for the strain *Schizothrix cf. delicatissima AMPL0116* that both RPS and sheath were mostly composed of macromolecules higher than 1 MDa.

EPS composition of the three cyanobacterial strains was characterized by a high complexity, and a high number of monosaccharides (up to 12, see Figure 8, Table 2, Supplementary Table S1) was identified. EPS composition of cyanobacterial sheaths and RPS showed different composition profiles. Within each EPS fraction, the composition was similar for *N. commune* and *P. ambiguum*, and different for *S. javanicum* (Figures 8A,B, Supplementary Table S1). The EPS released into the medium were mainly composed of glucose, and this monosaccharide was also the main component for *S. javanicum* and *P. ambiguum* sheaths (63.5 and 35.4%, respectively), while for *N. commune* the major sheath component was xylose (30.6%), and glucose as the second one (26.6%) (Figures 8A,B, Supplementary Table S1). This composition partially confirms the results of previous studies that reported the presence of different sugars in *N. commune* colonies such as glucose, galactose, xylose, and uronic acids (Helm et al., 2000). Hu et al. (2003) reported that glucose was the most abundant monosaccharide (44%) in *Nostoc* sp., with galactose and xylose being also found at a high molar percentage (21.5% and 20.9%, respectively). These authors also reported that most abundant monosaccharides in the EPS from *S. javanicum* were glucose (24.8%), galactose (23.4%) and mannose (22.9%), and for one species of the genus *Phormidium*, *P. tenue*, were arabinose (43.9%), glucose (32.5%) and rhamnose (10.4%).

### Growth of the Cyanobacterial Strains and Characteristics of the Induced EPS Matrix in the Sand Microcosms

A different pattern in cyanobacteria growth among the three strains was observed in the sandy soil microcosms. When inoculated on the sand, *S. javanicum* exhibited the highest growth, followed by *P. ambiguum*, as shown by the increase in chlorophyll content after one month of soil incubation (Figure 5). Both EPS fractions LB-EPS and TB-EPS increased over time as chlorophyll increased (Figure 6). A similar trend has been described in previous studies that documented an increase in chlorophyll *a* in cyanobacteria inoculated soils accompanied by an equivalent increase in EPS (Mazor et al., 1996; Chamizo et al., 2018). No growth was observed for *N. commune*, which showed a chlorophyll *a* content close to zero and similar to the non-inoculated sand. In their natural habitat, *N. commune* forms macroscopic colonies in which the entangled filaments are embedded in massive polysaccharidic structures which are crucial in the stress tolerance of this species to drought and frequent desiccation-rewetting (Potts, 1994) and freezing-thawing cycles (Tamaru et al., 2005). The chemical analysis on its RPS and sheath showed a higher percentage of low MW polymers (<50 kDa), compared to the two other strains, despite a very similar composition in monosaccharides to *P. ambiguum* EPS. The presence of smaller polymers may have reduced *N. commune* capability of forming stable aggregates in the sandy soil for growth sustain. Previous studies have shown the efficiency of this cyanobacterium to grow and lead to stable biocrusts over fine-textured soils (<70% sand) (Román et al., 2018; Roncero-Ramos et al., 2019a, b). However, in coarse soils, the ability of this cyanobacterium to bind sand grains and form stable organo-mineral layer could be reduced, as has been also reported for other cyanobacterial strains such as *Microcoleus vaginatus* (Rozenstein et al., 2014) or *Leptolyngbya ohadii* (Mugnai et al., 2020) on coarse sand. Of the two strains that showed a significant growth on the sandy soil, *S. javanicum* showed higher chlorophyll *a* (Figure 5) and LB-EPS contents than *P. ambiguum* (Figure 6A), regardless the latter strain was supplied with nitrogen-rich medium (BG11).

Under natural stressing conditions, cyanobacteria are considered to produce compositionally simpler EPS compared to liquid cultures, where they experience optimal abiotic conditions and excess of nutrients (Brüll et al., 2000). In the current study, under optimal conditions of water and nutrient availability, the soil EPS matrix was still characterized by a high number of monosaccharides (7–11), being almost always glucose largely the most abundant (Figure 8, Supplementary Table S1). While LB-EPS was compositionally simpler, TB-EPS showed a grater sugar diversity (Table 2) and besides glucose, galactose, mannose, xylose, and uronic acids were also relatively abundant. In induced biocrusts of different ages, mannose and glucose resulted the sugars present at the highest molar percentages in the LB-EPS fraction, while mannose, glucose, galactose and galacturonic acid were present at the highest molar percentages in the TB-EPS fraction (Chen et al., 2014). In inoculated sand with *S. javanicum* and *P. ambiguum*, but in the absence of nutrient supply and subjected to low water additions, the most abundant monosaccharides were glucose and galactose in the two soil EPS fractions, with also relatively high abundances of other sugars such as mannose, xylose, rhamnose and fructose in the TB-EPS fraction (Chamizo et al., 2019). According to Brüll et al. (2000), the heterogeneous sugar composition detected in our experiment could be due to the availability of nutrients. However, the experiment previously cited by Chamizo et al. (2019) led to a complex monosaccharidic profile even without nutrient addition, suggesting that in the natural conditions to which Brüll et al. (2000) refer, some other abiotic factors may influence the composition of the EPS.

When EPS features of the inoculated sandy soils were compared with the EPS profile of liquid cultures, we found that the soil TB-EPS fraction reflected both the MW distribution and monosaccharidic composition of the sheath but also the RPS fraction. Accordingly, Mugnai et al. (2018b) reported that EPS from the cyanobacterium *Schizothrix cf. delicatissima AMPL0116* was significantly different in soil growth compared to liquid cultures, both in composition and MW distribution, affecting these changes mainly the more condensed TB-EPS fraction, while no alterations were observed for the more soluble LB-EPS fraction. Their results showed that TB-EPS fraction was composed of a higher number of sugars than the LB-EPS fraction, and they attributed this difference to the different composition between sheath (composed of a higher number of sugar residues) and RPS (less heterogenous than the sheath). Our results, in turn, show that the TB-EPS fraction was very heterogeneous, resembling, on the contrary, the RPS fraction as witnessed by its high diversity compared to the sheath and by the presence of RPS-exclusive sugar residues both for *S. javanicum* and *P. ambiguum.* Moreover, the diversity describing the composition of the TB-EPS fraction resulted even higher than the RPS fraction alone (Table 2). It is indeed hard to dissect between two possible phenomena that may have occurred, that is, between the possibility that organisms produced polymers composed of different monosaccharides when growing on a different substrate, or that the complexity observed in TB-EPS composition was the result of the extraction procedure that removed a mixture of RPS and sheath EPS. The high diversity of TB-EPS leads us to believe that when the RPS get in contact with soil particles, the various chemical features of the sugars residues make the polymers bind tightly to the particles, so that they only become extractable with Na_2_-EDTA treatment (TB-EPS extraction), while only the very soluble glucose-based polymers can be extracted with water (LB-EPS extraction). This can easily be related to the fact that the growth and high EPS production capability of cyanobacteria in soil favor the formation of aggregates (Issa et al., 2001; Mager and Thomas, 2011).

### Implications for Soil Restoration

Production of EPS by cyanobacteria inoculation on the soil induces changes in soil properties important for a number of processes occurring at the soil surface. Cyanobacterial filaments together with their sticky EPS bind sand grains (Mugnai et al., 2018b), forming a cohesive and stable layer that contributes to reducing soil erosion (Kheirfam et al., 2017; Fattahi et al., 2020), one of the most important processes accelerating land degradation in drylands (Ravi et al., 2010). Experiments carried out in sand dunes in China have shown effective soil stabilization after inoculation with *Microcoleus vaginatus* and *S. javanicum*, encouraging soil colonization by other biocrust organisms and accelerating biocrust succession (Lan et al., 2014; Park et al., 2017). EPS also increase water retention capacity of the surface and thus play an important role in the maintenance of moisture (Mazor et al., 1996; Adessi et al., 2018). However, on very sandy soils, EPS have been also reported to retard water movement and reduce the amount of water that can penetrate into the sand (Mazor et al., 1996) thus decreasing hydraulic conductivity (Colica et al., 2014). We found that soil hydrophobicity was increased in the sandy soil inoculated with *S. javanicum*, probably associated to a higher growth and EPS release (especially of the LB-EPS) by this strain compared to *P. ambiguum*. In a previous study (Chamizo et al., 2019) we found no increase in hydrophobicity by inoculation of sandy soil with this strain possibly explained by the lower EPS amount (∼1 mg/g soil) under more water stressing conditions compared to the higher EPS amount (∼6.5 mg/g soil) recorded in this study where sandy soils were supplied with water and nutrients. Nevertheless, an increase in soil hydrophobicity by cyanobacteria inoculation on sandy soils could have as advantage a decrease in the time for runoff start and an increase in runoff yield, providing surplus water to downslope vegetation patches and favoring plant survival in drylands.

On the other hand, our results also showed that the monosaccharidic features of the released EPS and of sheath don’t represent *per se* an advantage for surviving in a sandy environment, as suggested by the scant growth of *N. commune*, despite the strain showed a polymer composition very similar to the EPS of *P. ambiguum*. Indeed, it might have been the smaller dimension of the secreted polymers that contributed in preventing *N. commune* growth in the sandy soil. These may be relevant information when planning the selection of cyanobacteria candidates for soil restoration purposes. In addition, our findings suggest that inoculation of the soil with the cyanobacterial biomass as well as their RPS can provide additional advantages derived from: (1) the supply of low MW molecules contained in the RPS fraction that could be easily hydrolyzed and used as carbon sources for soil heterotrophic microorganisms; (2) addition of a medium characterized by a diverse monosaccharidic composition, likely with different traits and contributing to increasing soil microbial heterogeneity. Indeed, in the attempt to extrapolate soil restoration techniques based on cyanobacteria inoculation from lab to the field, our findings point out the need of further considering the importance of the characteristics of the EPS released during culture growth in providing successful results, by improving conditions for cyanobacteria survival and growth and help them cope with abiotic stresses.

## Data Availability Statement

The raw data supporting the conclusions of this article will be made available by the authors, without undue reservation.

## Author Contributions

SC conceived the idea, designed the experiment, performed the laboratory analyses, analyzed the data, and wrote the manuscript. AA designed the experiment, performed the laboratory analyses, analyzed the data, and wrote the manuscript. GT designed the experiment, analyzed the data, and improved manuscript editing. RD conceived the idea, designed the experiment, and improved manuscript editing. All authors contributed with constructive comments to the manuscript.

## Conflict of Interest

The authors declare that the research was conducted in the absence of any commercial or financial relationships that could be construed as a potential conflict of interest.
