# Supplementary material for: Exopolysaccharide Features Influence Growth Success in Biocrust-forming Cyanobacteria, Moving From Liquid Culture to Sand Microcosms
Source: Front Microbiol. 2020 Oct 27;11:568224. doi: 10.3389/fmicb.2020.568224 (PMC7652855; doi:10.3389/fmicb.2020.568224)
Supplement: Supplementary file 1 [file Table_1.DOCX]

|  | | | **Monosaccharides (mol%)** | | | | | | | | | | | | |
| --- | --- | --- | --- | --- | --- | --- | --- | --- | --- | --- | --- | --- | --- | --- | --- |
| **Fraction** | **Strain** | **Growth**  **condition** | **Fuc** | **Rha** | **GalN** | **Ara** | **GlcN** | **Gal** | **Glc** | **Man** | **Xyl** | **Fru** | **Rib** | **GalA** | **GlcA** |
| RPS | *N.commune* | liquid | 1.54 | 2.29 | *n.d.* | 2.99 | 1.8 | 8.51 | **26.59** | **19.3** | **30.64** | 0.79 | 0.38 | 1.22 | 3.97 |
| RPS | *S.javanicum* | liquid | 5.93 | 0.36 | *n.d.* | 2.78 | *n.d.* | 7.43 | **63.52** | *n.d.* | **16.37** | *n.d.* | *n.d.* | 1.67 | 1.95 |
| RPS | *P.ambiguum* | liquid | 1.27 | 1.18 | *n.d.* | 3.36 | *n.d.* | 8.55 | **35.43** | **24.29** | **18.57** | 0.98 | 0.71 | 2.15 | 3.51 |
| Sheath | *N.commune* | liquid | 1.27 | 3.64 | *n.d.* | 1.37 | 0.91 | 1.03 | **88.74** | *n.d.* | *n.d.* | *n.d.* | 0.7 | 0.37 | 0.42 |
| Sheath | *S.javanicum* | liquid | 1.14 | 2.89 | *n.d.* | 0.91 | 0.48 | 5.34 | **65.35** | **10.26** | *n.d.* | 5.46 | 7.51 | 0.41 | 0.26 |
| Sheath | *P.ambiguum* | liquid | 1.26 | 3.63 | *n.d.* | 1.37 | 0.9 | 1.03 | **88.37** | *n.d.* | *n.d.* | 1.97 | 0.7 | 0.36 | 0.42 |
| LB-EPS | *S.javanicum* | sand | 1.86 | 3.24 | *n.d.* | 1.74 | *n.d.* | **14.13** | **72.96** | *n.d.* | *n.d.* | *n.d.* | 4.21 | 0.84 | 1.03 |
| LB-EPS | *P.ambiguum* | sand | 0.43 | 1.45 | *n.d.* | 0.63 | 0.19 | 1.85 | **93.99** | *n.d.* | *n.d.* | 0.67 | 0.59 | *n.d.* | 0.21 |
| TB-EPS | *S.javanicum* | sand | 2.96 | 2.51 | *n.d.* | 1.41 | 0.95 | **12.96** | **35.34** | **15.14** | **17.81** | *n.d.* | *n.d.* | 5.78 | 5.16 |
| TB-EPS | *P.ambiguum* | sand | 0.45 | 0.12 | 1.83 | 0.92 | 1.79 | **16.56** | **35.84** | **22.61** | **10.89** | 1.25 | *n.d.* | 2.25 | 5.52 |

Table S1. Monosaccharidic composition of the extracted polysaccharides, for each strain, each fraction and each growth condition. Data show the molar percentages (%) of single sugars (expressed as moles of the single monosaccharide divided by the total amount of moles of monosaccharides in the EPS × 100). Bold numbers indicate the most represented sugars (>10%). No data are reported for *N. commune* in the sandy soil microcosms because of the negligible amounts of EPS extracted. Abbreviations: n.d. not detectable; Fuc fucose, Rha rhamnose, GalN galactosamine, Ara arabinose, GlcN glucosamine, Gal galactose, Glc glucose, Man mannose, Xyl xylose, Fru fructose, Rib ribose, GalA galacturonic acid, GlcA glucuronic acid.
